# Oxidation-induced thermopower inversion in nanocrystalline SnSe thin film

**DOI:** 10.1038/s41598-021-81195-7

**Published:** 2021-01-15

**Authors:** Sunao Shimizu, Kazumoto Miwa, Takeshi Kobayashi, Yujiro Tazawa, Shimpei Ono

**Affiliations:** 1 Materials Research Laboratory, Central Research Institute of Electric Power Industry (CRIEPI), Yokosuka, Kanagawa 240-0196 Japan; 2 Electric Power Engineering Systems, Yokosuka, Kanagawa 240-0101 Japan

**Keywords:** Materials science, Thermoelectric devices and materials

## Abstract

Given the growing demand for environmentally friendly energy sources, thermoelectric energy conversion has attracted increased interest as a promising CO_2_-free technology. SnSe single crystals have attracted attention as a next generation thermoelectric material due to outstanding thermoelectric properties arising from ultralow thermal conductivity. For practical applications, on the other hand, polycrystalline SnSe should be also focused because the production cost and the flexibility for applications are important factors, which requires the systematic investigation of the stability of thermoelectric performance under a pseudo operating environment. Here, we report that the physical properties of SnSe crystals with nano to submicron scale are drastically modified by atmospheric annealing. We measured the Seebeck effect while changing the annealing time and found that the large positive thermopower, + 757 μV K^−1^, was completely suppressed by annealing for only a few minutes and was eventually inverted to be the large negative value, − 427 μV K^−1^. This result would further accelerate intensive studies on SnSe nanostructures, especially focusing on the realistic device structures and sealing technologies for energy harvesting applications.

## Introduction

Thermoelectric energy conversion has recently become an area of increased interest as a promising technology for generation of renewable energy^1–3^, as part of the urgent need to develop a carbon neutral society^4–6^. In the search of thermoelectric materials for practical applications, many studies have focused on doped semiconductors such as Bi_2_Te_3_ and PbTe^2,7^. However, those compounds are based on harmful heavy elements and lack heat and acid resistance, resulting in various problems such as toxicity to human, environmental pollution, and high costs for production and recycling. One category of alternatives to these classic thermoelectric materials is layered metal chalcogenide^8–14^. The two-dimensional structures have recently gained much attention owing to their unique properties such as enhanced thermoelectric response based on peculiar electronic properties^15,16^ and greatly-suppressed thermal conductivity arising from anisotropic crystal structures^2^.

Among the two-dimensional layered chalcogenides, SnSe has attracted a considerable interest, demonstrating chemical stability and low toxicity^17–20^. As well, it should be noted that an extremely low lattice thermal conductivity is realized, which allows SnSe to possess record-high *ZT* values at high temperatures. For example, the *ZT* values larger than 2 were demonstrated for both *p*-type^17^ and *n*-type^19^ SnSe at high temperatures; these studies have revealed the significant potential of single-crystalline SnSe as an excellent bipolar thermoelectric material. The next challenge is, therefore, the systematic investigation of polycrystalline SnSe from multiple viewpoints in order to harness SnSe for practical applications. The use of polycrystalline SnSe would be required due to the flexibility of application and the lower production costs.

SnSe displays a high thermoelectric performance at temperatures above around 700 K regardless of single crystals or nanocrystals^17,19,21,22^ and thus would be used in a high temperature region for energy harvesting. Here, the problem is that the surface of SnSe crystals is quickly oxidized when exposed to oxygen at high temperatures^23,24^. When evaluating the intrinsic thermoelectric properties of SnSe, such oxidation has been carefully avoided during synthesis and experimental proceedures^17,19,21,25^. Actually, the effect of oxidation on thermoelectric performance of SnSe has been carefully investigated focusing on the thermal conductivity^26^. In contrast, however, there are not so many studies on how surface oxidation affects the Seebeck effect of SnSe^27–29^. In particular, in nano- to submicron-scale crystals, the large surface to volume ratio would end up emphasizing the surface contribution to physical and chemical properties; as such, the high temperature operation can have a serious impact on thermoelectric modules using SnSe. It is therefore necessary to evaluate the stability of the electronic and thermoelectric properties of SnSe, assuming a realistic operating environment.

Here, we report that the physical properties of nanocrystalline SnSe thin films are drastically modified by atmospheric annealing. In order to evaluate the development of the surface oxidation, the systematic measurements of optical and thermoelectric properties were performed with changing the annealing temperature and time; principal properties of semiconductors such as the band gap, the chemical potential, and the polarity of the charge carriers are sensitively monitored by those measurements. It was found that the large positive thermopower, + 757 μV K^−1^, of the SnSe thin films was completely suppressed by annealing for only a few minutes and even showed the sign inversion. This result would further accelerate intensive studies on SnSe nanostructures, especially focusing on the realistic device structures and sealing technologies for energy harvesting applications.

## Results and discussion

**Figure 1:**
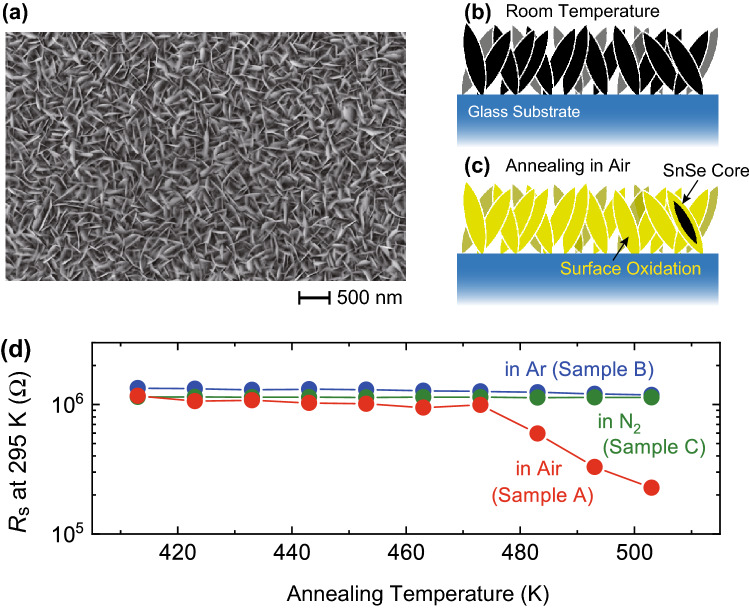
Atmospheric annealing on SnSe thin films. (**a**) Scanning electron microscopy (SEM) of thermally evaporated SnSe thin films. A web-like structure with a nano- to submicron-scale comprises the thin films^25^. (**b**) Schematic image of SnSe nanostructure on glass substrate. (**c**) Schematic image of surface oxidation of SnSe nanostructure. An oxide layer is formed on the surface of SnSe when SnSe is exposed to air or oxygen at high temperatures^23,24^. (**d**) Modulation of sheet resistance *R*_s_ by atmospheric annealing. The three SnSe thin films, Samples A, B, and C, were annealed for 30 min at each annealing temperature. The values of *R*_s_ at 295 K decreased by annealing in air, while they did not change in Ar and N_2_ atmosphere. The image (**a**) was created by using Hitachi PC-SEM (Version 09-05-0932), which is a software to control SEM (Hitachi S-4300SE/N).

In order to focus on the surface oxidation effect, we adopted SnSe thin films fabricated by thermal evaporation, following the procedure described elsewhere^25^. This is because the thermally-evaporated SnSe thin films compose of porous nanosheets networks with nano to submicron scales^25^, as shown in a scanning electron microscopy (SEM) image in Fig. 1a, and the large surface area would enable us to sensitively monitor the development of oxidation. Figure 1b,c schematically illustrates a side view of the SnSe thin film in its initial state and that with surface oxidation layer after high temperature annealing.

The surface oxidation was clearly observed in the form of a drastic decrease in the electrical resistance. We measured the sheet resistance *R*_s_ of the SnSe thin films for different annealing conditions, as shown in Fig. 1d. The three SnSe thin films, Samples A, B, and C, were annealed for 30 min at each annealing temperature, and *R*_s_ were evaluated at 295 K after cooling from the annealing temperatures. We started the annealing at 413 K (140 ℃) and increased the annealing temperature up to 503 K (230 ℃). Sample A was annealed in an atmospheric condition, where the relative humidity was 40% at 25 ℃, on a digital hotplate. As shown in Fig. 1d, *R*_s_ at 295 K decreased when the annealing temperature was higher than ~ 473 K, and decreased further as the annealing temperature was increased. Samples B and C were annealed in globe boxes filled with Ar and N_2_ gas, respectively, where the oxygen level was lower than 0.1 ppm and the dew point temperature was lower than − 80 ℃. The values of *R*_s_ at 295 K were not affected by annealing in the measured temperature range. This result suggests that the reduction of *R*_s_ seen in Fig. 1d is not related to a possible Se deficiency during annealing process but attributed to an oxygen exposure at high temperatures. If the reduction of *R*_s_ originates from the Se deficiency induced at high temperatures, such a reduction should be observed in Ar and N_2_ atmospheres as well. In all the other experiments described here, the annealing was performed in air at a fixed temperature, 483 K (210 ℃), in order to focus on the annealing time dependence of physical properties.

**Figure 2:**
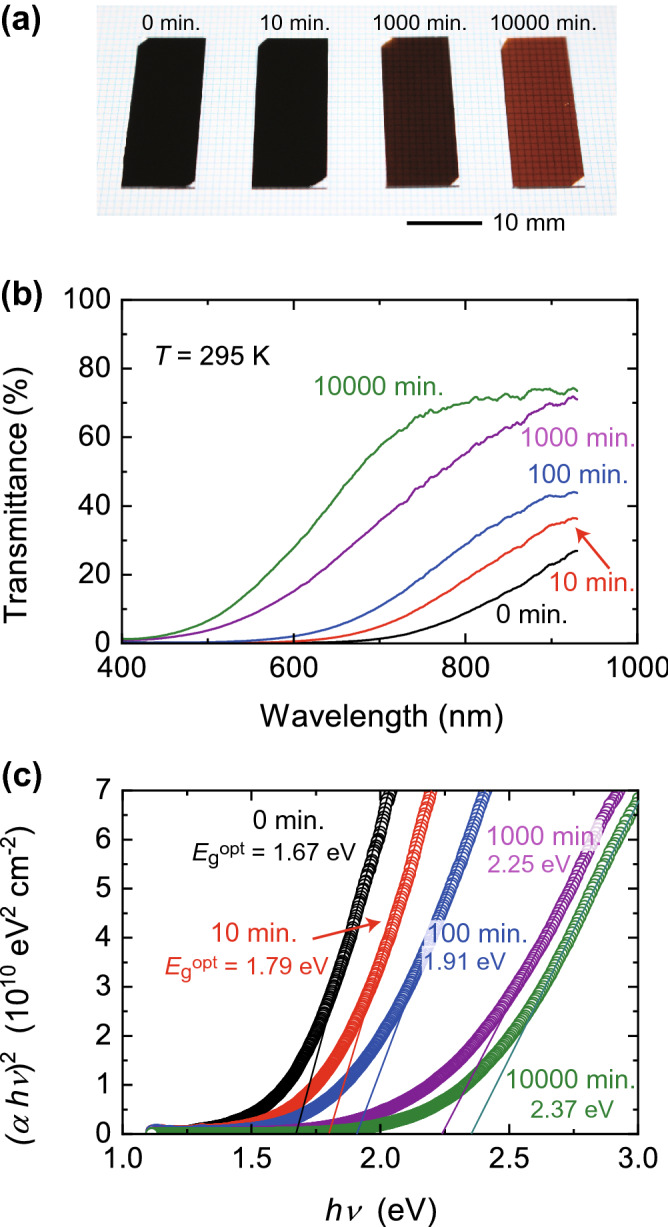
Modulation of optical band gap in SnSe thin films. (**a**) Photographs of SnSe thin films annealed at 483 K (210 ℃) for different annealing times. The color of the thin films gradually changed with an increase in the annealing time. The thin films became transparent when the annealing time reached 10,000 min. (**b**) Transmittance of SnSe thin films. The transmittance for the visible light region increased with an increase in the annealing time, following the same trend as the color change shown in (**a**). The temperature *T* for the measurement was 295 K. (**c**) Estimation of optical band gap *E*_g_^opt^ of SnSe thin films following Tauc plot. The values of *E*_g_^opt^ were determined by extrapolating the linear region of the (*αhν*)^2^ vs. *hν* plot to the *x* axis, where *α* is absorption coefficient and *hν* is photon energy.

The optical response of the SnSe thin films also changed dramatically due to the surface oxidation. Figure 2a shows a photograph of the SnSe thin films for different annealing times. For comparison of the transparency, the samples were placed on a paper with lines of a 1 mm spaced grid pattern. The SnSe film looked completely black at the initial state, which is denoted as 0 min, and became gradually transparent with increasing the annealing time. The grid lines were seen through the thin film and the glass substrate when the annealing time reached 10,000 min. Figure 2b shows the transmittance of SnSe thin films in the wavelength region that includes visible light. The visible light transmission of the non-annealed SnSe was effectively inhibited with the transmittance lower than ~ 10%, which is consistent with previous research^30–32^. However, the transmittance increased with increasing annealing time, which is the same trend as the color change of the SnSe thin films in Fig. 2a. In addition, we performed a control experiment to evaluate how the transmittance of a non-annealed SnSe thin film changes for different air exposure time at room temperature. Supplementary Figure S1 reveals that the transmittance did not show a meaningful change when the sample was kept in air at room temperature. This suggests that the optical band gap *E*_g_^opt^ is widened due to the annealing-induced oxidation and thus inhibits the absorption of the visible light.

The values of *E*_g_^opt^ are obtained from a relationship between the absorption coefficient *α* and a photon energy *hν* following the Tauc relation^33^,

1 $$(\alpha h \nu) = {\text{ B}}(h \nu - E_{{\text{g}}}{^{{{\text{opt}}}}} )^{{\text{n}}} ,$$

where *h* is the Planck constant, *ν* is the frequency of light, and B is a constant. The value of n is 0.5 for direct transitions and 2 for indirect transitions. Figure 2c shows the (*αhν*)^2^ vs. *hν* plot for the SnSe films, where *α* was estimated from the transmittance according to the Lambert law^34^. The linear relation of (*αhν*)^2^ and *hν* was confirmed, as demonstrated by the solid lines, suggesting the direct transition nature of the SnSe films. The values of *E*_g_^opt^ were determined by extrapolating the linear region of the (*αhν*)^2^ vs. *hν* plot to the *x* axis, as shown in Fig. 2c. For the initial SnSe thin film, an indirect *E*_g_^opt^ was estimated to be 1.67 eV, which is comparable to preceding studies of SnSe thin films but larger than that of bulk^35^. With increasing the annealing time, *E*_g_^opt^ monotonically increased up to 2.37 eV.

**Figure 3:**
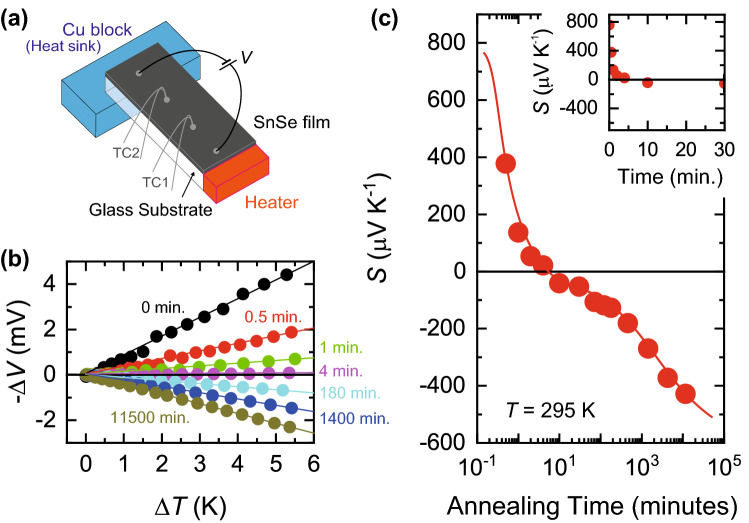
Thermopower inversion induced by surface oxidation. (**a**) Schematic sample configuration for thermoelectric measurements. A heater and a Cu block were attached to the SnSe thin films to induce a temperature gradient along the longer direction. The two thermocouples, TC1 and TC2, were used to record the thermoelectric voltage Δ*V* and the temperature difference Δ*T*. An input voltage *V* was applied when the four-terminal resistance of the films was measured. (**b**) Δ*V* − Δ*T* plot of SnSe thin films for different annealing time. The values of Δ*V* linearly increased with Δ*T*, assuring that the thermoelectric effect was correctly measured. (**c**) Seebeck coefficient *S* as a function of annealing time. The temperature *T* for the measurement was 295 K. The values of *S* were drastically modified by annealing, where the total change of *S* was as large as ~ 1200 μV K^−1^. The solid line is a guide to the eye. The inset is the expansion of the data for 0 to 30 min of annealing.

Thermoelectric response is a sensitive tool to detect the modulation of the electronic structures because it is closely related to physical properties such as the band gap, the Fermi energy, and the polarity of transport carriers^36^. Figure 3a schematically shows the sample configuration for thermoelectric measurements (see “Experimental section”). Two thermocouples, TC1 and TC2, measured the thermoelectric voltage Δ*V* under the temperature difference Δ*T*. Figure 3b shows the Δ*V* − Δ*T* plot of the SnSe thin films for different annealing times. The values of Δ*V* increased linearly with Δ*T*, indicating that the thermoelectric effect was correctly measured. It is noted that, surprisingly, the sign of the thermoelectric response changed as the annealing time was increased. The sign of Δ*V* corresponds to the polarity of the transport carriers^36^, suggesting that the dominant carriers changed from holes to electrons. Figure 3c shows the transition of the Seebeck coefficient *S* as a function of annealing time. The initial SnSe film without annealing has a large positive *S*, + 757 μV K^−1^, as shown in the inset of Fig. 3c. By annealing the sample for only a few minutes in air, the large thermoelectric effect was completely suppressed. Further annealing negatively increased *S*, which reached − 427 μV K^−1^ when the annealing time was 11,500 min. The oxidation induced a drastic thermopower inversion, where the total change of *S* was as large as ~ 1200 μV K^−1^.

**Figure 4:**
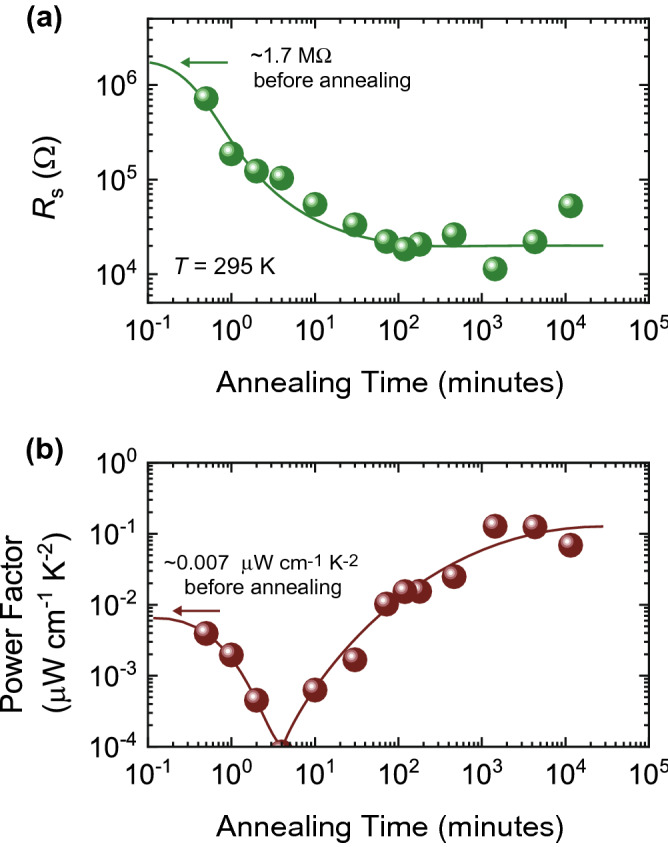
Evaluation of power factor in SnSe thin films annealed in air. (**a**) Sheet resistance *R*_s_ of SnSe thin films as a function of annealing time. The temperature *T* for the measurement was 295 K. The values of *R*_s_ decreased down to ~ 10^4^ Ω, which was 100 times smaller than the initial state, with an increase in the annealing time. (**b**) Evolution of power factor of SnSe. The power factor is defined as *S*^2^/(*R*_s_ × *d*), where *S* and *d* are the Seebeck coefficient and the film thickness, respectively. The power factor seems to reach a final value of ~ 0.1 μW cm^−1^ K^−2^ at around 10^4^ min, which is comparable to the room temperature value reported for SnO_2_ in previous studies^52–56^. The solid lines are guides to the eye.

When SnSe thin films are annealed in air, SnO_2_ would begin to form on the surface of SnSe^23,24^. SnO_2_ is a prototypical wide band gap semiconductor and shows an excellent transparency for visible light^37,38^. The values of *E*_g_^opt^ of the SnSe thin films increased up to 2.37 eV, as shown in Fig. 2c. This value is smaller than 3.6 eV for pure SnO_2_ but comparable to 2.3 eV for impurity-doped SnO_2_^39^, suggesting that the atmospheric annealing changed the surface of SnSe to SnO_2_. In order to further investigate the oxidation process, we performed SEM, EDX, and XRD analysis (see Supplementary Note). Supplementary Figures S2 and S3 show the SEM images and the EDX analysis, respectively, for different annealing time. It was found that the oxidation developed with increasing the annealing time, even though the morphology of the SnSe thin films did not change. The XRD spectra in Supplementary Figs. S4 and S5 demonstrate the oxidation process in more detail; first, SnO_2_ was formed, and then another *n*-type semiconductor SnSe_2_^40,41^ was developed, especially when the annealing time exceeded ~ 1000 min. Figure 4a shows *R*_s_ for the SnSe thin films as a function of annealing time. The values of *R*_s_ decreased down to ~ 10^4^ Ω, which is 100 times smaller than the initial state, as the annealing time was increased. The reduction of *R*_s_ is attributed to the gradual formation of SnO_2_ and SnSe_2_; they can possess high electrical conductivity due to the large electron mobility at room temperature^37,42,43^ when electrons are doped by oxygen deficiency or impurity. The thermoelectric response reflects the oxidation clearly because the sign of the thermoelectric response is different for *n*-type and *p*-type semiconductors. The values of the power factor *S*^2^/(*R*_s_ × *d*), where *d* is the thickness of the thin films, show the continuous change, as seen in Fig. 4b, reflecting the ratio of contributions from SnSe and the oxidation layer. With increasing the annealing time, *S*^2^/(*R*_s_ × *d*) dropped to zero and increased up to a saturated valued of ~ 0.1 μW cm^−1^ K^−2^, suggesting that the oxidation layer eventually dominates the electronic properties of the thin films; high conducting components dominate in thermoelectric response in parallel conduction pathways^44^.

The atmospheric annealing effect investigated here suggests the importance of well-designed device architectures for real-world applications. In this study, we annealed the SnSe thin films at relatively low temperatures of around 480 K and found significant modification of physical properties. In practical applications, SnSe would be utilized at temperatures higher than 480 K because the thermoelectric performance of SnSe is optimized in the high temperature region above 700 K^17–19,22,45^, which is far above the annealing temperature adopted in this study. Moreover, the size of the SnSe nanostructures composing the thin film, as shown in Fig. 1a, is in the order of 10 nm to submicron; this scale is comparable with the previously studied nanocrystalline SnSe^25,46^ or the polycrystalline grains in SnSe pellets and sintered samples for thermoelectric applications^22,27,28,47–49^. This means that the annealing effect investigated in the current study would also occur in thermoelectric devices based on SnSe. A recent study showed that a careful removal of the surface layer on SnSe crystals enhanced the thermoelectric performance^28^. SnSe has shown record-high *ZT* values and there is no doubt of its potential for the energy harvesting applications in the near future. Therefore, designing realistic system packages and developing various sealing technologies would make an important contribution to the development of next-generation thermoelectric devices.

## Conclusions

Our results provide important implications on the strategies to utilize an ideal high-temperature thermoelectric material, SnSe. We fabricated nanocrystalline SnSe thin films by thermal evaporation and investigated the effect of annealing on thermoelectric performance. It was found that the physical properties of the SnSe thin films were dramatically modified by atmospheric annealing at a relatively low temperature of around 480 K. With an increased annealing time, the transparency to visible light increased, while *S* changed its sign from positive to negative. Surprisingly, the thermopower was modulated by atmospheric annealing, showing even the sign inversion from + 757 to − 427 μV K^−1^. This indicates that the atmospheric annealing induced surface oxidation that formed n-type semiconductors, which finally dominated the physical properties of the thin films. This study further expands the range of intensive studies on SnSe nanostructures, especially focusing on realistic device structures and sealing technologies for energy harvesting applications. The multifunctional nature of SnSe, including active layers in photovoltaics^35,50^ and electrode materials in secondary batteries^51^, can play important roles in producing renewable energy essential in future.

## Experimental section

### Sample preparation

The SnSe thin films with the thickness of 0.5 μm were synthesized following the procedure described elsewhere^25^ on glass substrates (Corning Eagle XG) having a root-mean-square for surface roughness of less than 1.5 nm, which was purchased from Corning Incorporated. The physical and chemical analyses of thermally evaporated SnSe were reported in detail elsewhere^25^. It is expected that the surface of the SnSe nanosheets are oxidized at high temperatures^23,24^, as schematically shown in Fig. 1b,c. The SnSe thin films were annealed under different conditions, which is discussed in “Results and discussion” in detail.

### Thermoelectric measurements

The typical size of the glass substrate used for the thermoelectric measurements was 4 mm × 7 mm × 0.7 μm. As shown in Fig. 3a, a heater and a heat sink were attached to either side of the sample to produce a thermal gradient. The type E thermocouples were attached to monitor the temperature difference Δ*T* and the thermoelectric voltage Δ*V*. The thermocouples were also used for the four-terminal measurements of *R*. The temperature difference Δ*T* and the voltage Δ*V* between the thermocouples were measured, and the values of *S* were evaluated from the slope of the Δ*V *− Δ*T* plots (see Fig. 3b). This sample configuration allows us to measure *S* and *R*_s_ simultaneously.

## Supplementary Information

Supplementary Information.
