# Supplementary material for: Oxidation-induced thermopower inversion in nanocrystalline SnSe thin film
Source: Sci Rep. 2021 Jan 15;11:1637. doi: 10.1038/s41598-021-81195-7 (PMC7810839; doi:10.1038/s41598-021-81195-7)
Supplement: Supplementary file 1 — Supplementary Information. [file 41598_2021_81195_MOESM1_ESM.pdf]

Supplementary Information for
"Oxidation-induced thermopower inversion in nanocrystalline SnSe thin film"

Sunao Shimizu[1,*], Kazumoto Miwa[2], Takeshi Kobayashi[1], Yujiro Tazawa[1], and Shimpei Ono[1]

[1]Materials Research Laboratory, Central Research Institute of Electric Power Industry (CRIEPI), Kanagawa, 240-0196, Japan

[2]Electric Power Engineering Systems, Kanagawa, 240-0101, Japan

[*]s-sunao@criepi.denken.or.jp

Supplementary Notes

SEM, EDX, and XRD analysis of SnSe thin films for different annealing time

Supplementary Figure S2 shows the SEM images for different annealing time. The annealing was done at 483 K. When we increase the annealing time from 0 minute (Supplementary Fig. S2a) to 10950 minutes (Supplementary Fig. S2e), the morphology of the thin films did not show noticeable changes. On the other hand, however, EDX spectra systematically changed with increasing the annealing time. Supplementary Figures S3a-S3e show the EDX spectra of the SnSe thin films for different annealing time. Supplementary Figure S3a is the EDX for the non-annealing sample, the result of which is consistent with the previous study[1]. In addition to the signal from Sn and Se, we observed Si, O, and C. The small peaks for Si and O would originate from the glass substrate, while the high level of C indicates the contamination from the environment. In Supplementary Figs. S3f-S3j, the ratio of Sn, Se, Si, and O is shown, where the oxygen level increases with increasing the annealing time. This suggests that the oxidation proceeds with increasing the annealing time, even though the morphology of the SnSe thin films does not change.

Supplementary Figures S4 and S5 show the XRD spectra of SnSe thin films for different annealing time. Before annealing (0 minunte), the diffraction peaks similar to previous studies of SnSe[2–7] were obtained. With increasing the annealing time, the profile of peaks around $2\theta \sim 31°$ was continuously changed. Supplementary Figure S4b shows the magnified view around $2\theta \sim 31°$. When the SnSe thin film was annealed for 10 minutes, the peak for SnO_2 emerged. The peaks were merged into a broad single

peak at 1000 minutes. Finally, at 10000 minutes, the peak position moved to $2\theta \sim 30.4°$, which corresponds to an *n*-type semiconductor $SnSe_2$. The transition of the XRD profile suggests that the annealing process develops as follows. First, SnO_2 layer emerges on the surface of SnSe. Then $SnSe_2$ is formed due to the lack of Sn, especially when the annealing time exceeds ~1000 minutes.

Importantly, the development of EDX and XRD shown in Supplementary Figs. S3 and S4 is consistent with the modulation of the Seebeck coefficient S in Fig. 3c. With the progress of the oxidation, S was suppressed and showed the sign change. In addition, the annealing time dependence of S in Fig. 3c changed its slope at around 500 minutes, which would correspond to the change of the XRD spectra around 1000 minutes in Fig. S4.

SEM and EDX were performed on Hitachi S-4300SE/N with EDAX EDX attachment. XRD was performed on Bruker D8 diffractometer with Cu Kα radiation.

Supplementary Figures

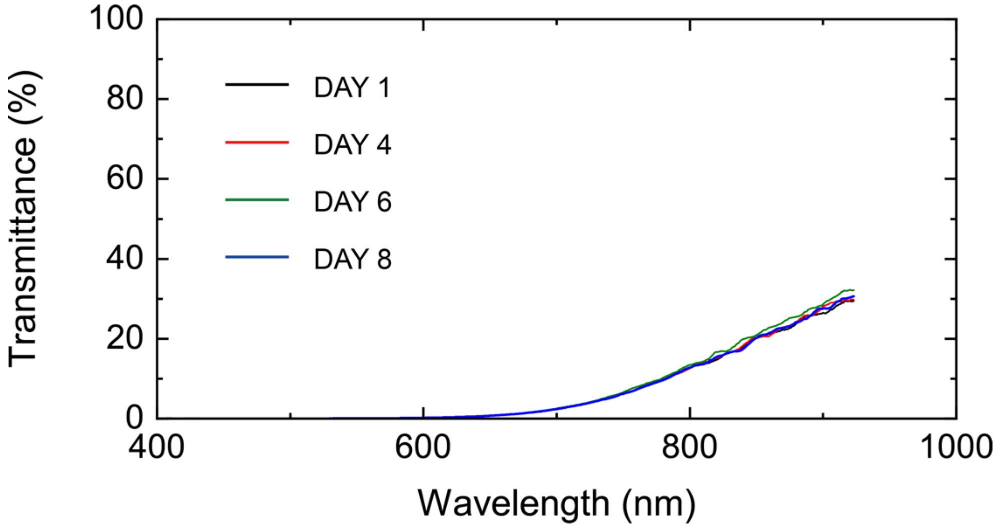

Supplementary Figure S1. Transmittance of SnSe thin film for different air exposure time at 295 K. We kept the thin film in air at 295 K to investigate whether the change of the transmittance in Fig. 2b is solely attributed to the annealing at elevated temperatures. Here, DAY n means the n-th day since the start of the air exposure. It was confirmed that the transmittance did not show a meaningful change when the sample was kept in air at 295 K.

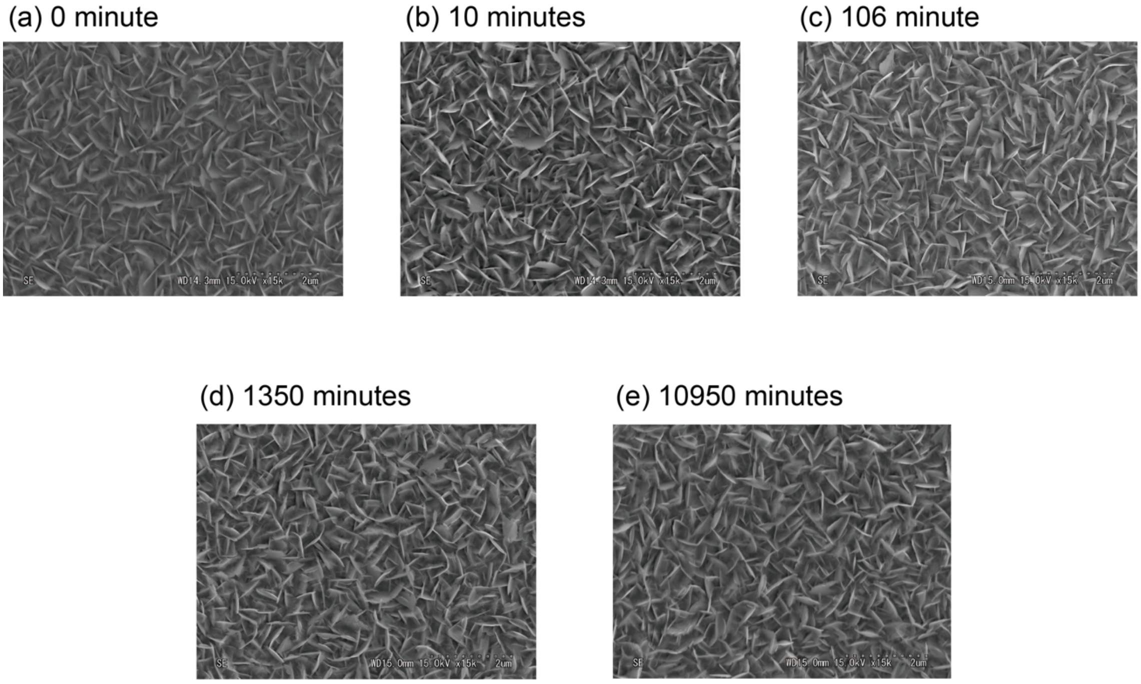

Supplementary Figure S2. Scanning electron microscopy (SEM) of thermally evaporated SnSe thin films for different annealing time. The annealing was done at 483 K. The images were created by using Hitachi PC-SEM (Version 09-05-0932, the URL for the software does not exist), which is a software to control SEM (Hitachi S-4300SE/N).

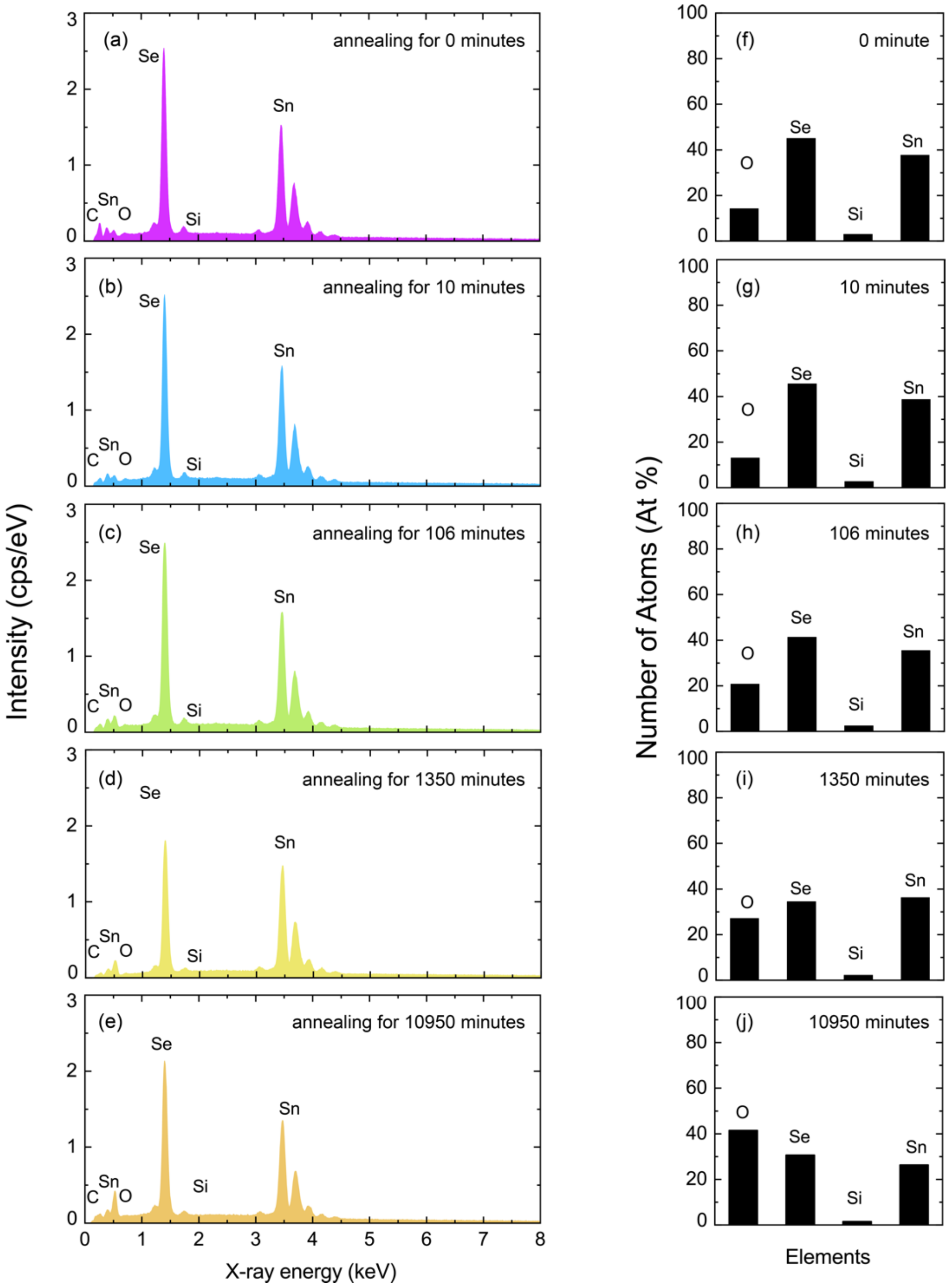

Supplementary Figure S3. SEM-EDX analysis of SnSe thin films for different annealing time. (a-e) EDX spectra for SnSe thin films. The ratio of O against Se and Sn increases as shown in (f-j).

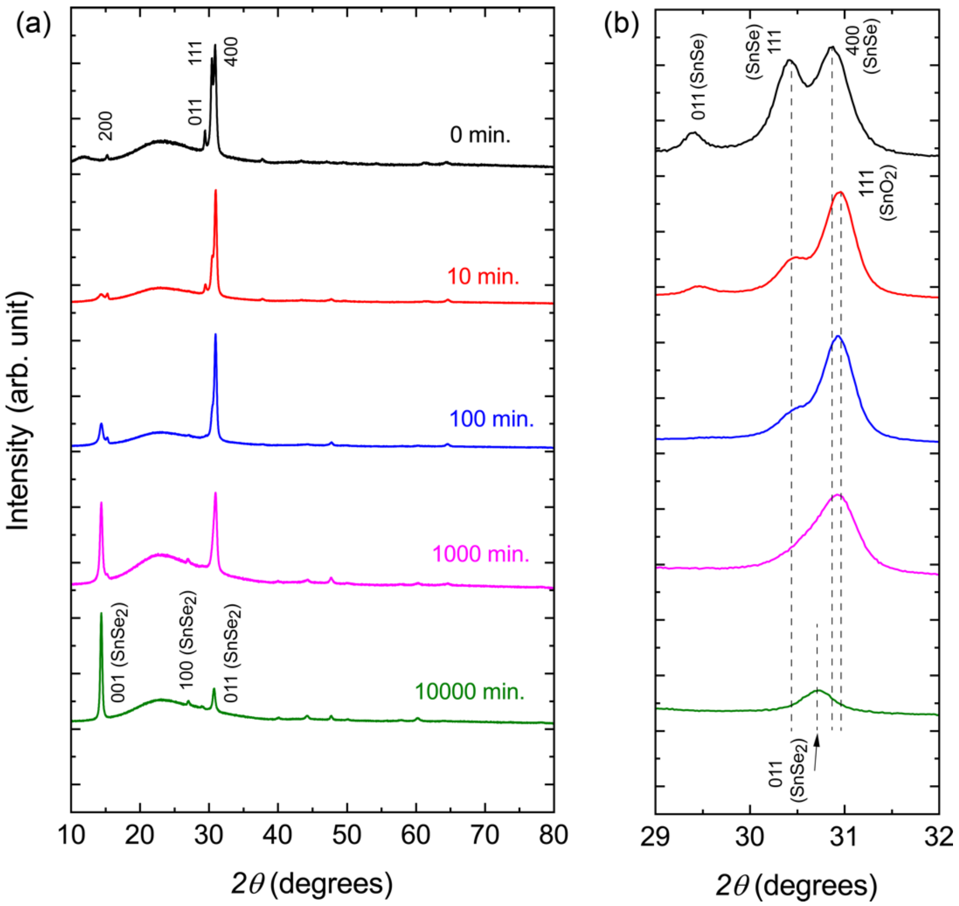

Supplementary Figure S4. XRD spectra of SnSe thin films for different annealing time. (a) With increasing the annealing time, the peaks around 14° and 31° were gradually changed. (b) Magnified view of XRD spectra in (a) around $2\theta \sim 31°$. See Supplementary Fig. S5 for the peak assignment.

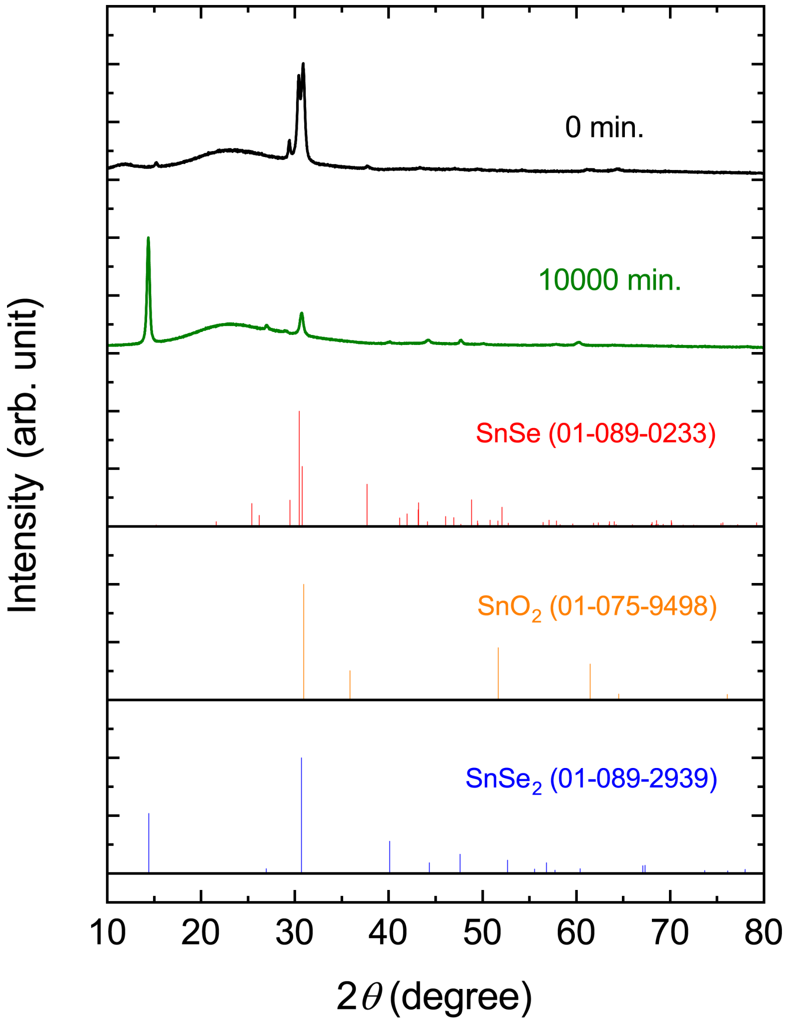

Supplementary Figure S5. Peak assignment of XRD patterns. The experimental data for 0 minute and 10000 minutes are same with those in Fig. S4. We used the PDF files of #01-089-0233, #01-0890-9233, and #01-089-0293 for the peak assignment of SnSe, SnO_2, and $SnSe_2$, respectively. The PDF files are obtained from the database of International Centre for Diffraction Data (ICDD).

Supplementary Reference

1. Burton, M. R. *et al.* Thin Film Tin Selenide (SnSe) Thermoelectric Generators Exhibiting Ultralow Thermal Conductivity. *Adv. Mater.* **30**, 1801357 (2018).
2. Biçer, M. & Şişman, I. Electrodeposition and growth mechanism of SnSe thin films. *Appl. Surf. Sci.* **257**, 2944–2949 (2011).
3. Kumar, N. *et al.* Structure, Optical and electrical characterization of tin selenide thin films deposited at room temperature using thermal evaporation method. *J. Nano- Electron. Phys.* **3**, 117–126 (2011).
4. Im, H. S. *et al.* Facile phase and composition tuned synthesis of tin chalcogenide nanocrystals. *RSC Adv.* **3**, 10349–10354 (2013).
5. Ariswan, Sutrisno, H. & Prasetyawati, R. Crystal Structure, Optical, and Electrical Properties of SnSe and SnS Semiconductor Thin Films Prepared by Vacuum Evaporation Techniques for Solar Cell Applications. *IOP Conf. Ser. Mater. Sci. Eng.* **202**, 012042 (2017).
6. Reddy, V., Reddy, M., Lindwall, G., Pejjai, B. & Gedi, S. Solar Energy Materials and Solar Cells α -SnSe thin fi lm solar cells produced by selenization of magnetron sputtered tin precursors. *Sol. Energy Mater. Sol. Cells* **176**, 251–258 (2018).
7. Lee, Y. K., Luo, Z., Cho, S. P., Kanatzidis, M. G. & Chung, I. Surface Oxide Removal for Polycrystalline SnSe Reveals Near-Single-Crystal Thermoelectric Performance. *Joule* **3**, 719–731 (2019).
